# Consumers' knowledge, attitude, and behavior towards antimicrobial resistance and antimicrobial use in food production in China

**DOI:** 10.3389/fpubh.2022.1015950

**Published:** 2022-09-23

**Authors:** Qianyun Ding, Jiuzhi Gao, Xianfeng Ding, Dan Huang, Yunfeng Zhao, Min Yang

**Affiliations:** ^1^Department of 'A', The Children's Hospital, Zhejiang University School of Medicine, National Clinical Research Center for Child Health, Hangzhou, China; ^2^National Institute for Nutrition and Health, Chinese Center for Disease Control and Prevention, Beijing, China; ^3^College of Life Sciences and Medicine, Zhejiang Sci-Tech University, Hangzhou, China; ^4^Department of Big Data in Health Science, School of Public Health, Zhejiang University School of Medicine, Hangzhou, China; ^5^Center of Clinical Big Data and Analytics of the Second Affiliated Hospital, Zhejiang University School of Medicine, Hangzhou, China; ^6^Department of Nutrition and Food Hygiene, School of Public Health, Zhejiang University School of Medicine, Hangzhou, China

**Keywords:** antimicrobial resistance (AMR), antimicrobial use (AMU), food production, consumer, knowledge-attitude-behavior, China

## Abstract

**Background:**

Antimicrobial resistance (AMR) can be induced by overuse or misuse of antimicrobials. Few researches were involved in consumers' knowledge and attitude toward antimicrobial use (AMU) in food production. This study was designed to investigate the knowledge and awareness, perception, and attitude of Chinese consumers toward AMU in food production. Their behavior, purchase intention of antimicrobial-free food products, and confidence in information sources were also investigated.

**Methods:**

As a descriptive cross-sectional study, an online electronic survey questionnaire was conducted between February 25 and March 8, 2022, involving 1,065 consumers in China. Factor analysis was conducted to identify underlying patterns of the attitudes and information sources. Spearman correlations were employed to determine the relationship between knowledge, attitudes and the intention to pay extra. The differences in knowledge and attitudes were performed by independent *t*-test and one-way analysis of variance (ANOVA) test, and the difference in intention was performed by Chi-square test, when compared with demographic factors.

**Results:**

The findings showed that even though 75.0% of them heard of AMR, and 48.2% knew the definition of AMR, the level of consumers' knowledge of AMU in farming production and food regulations in China was not high (48.9% of participants replied correctly). About half viewed AMU and AMR as a potential risk to their health. Of these participants, 61.3% claimed that they were more likely looking for specific information about AMU on food packaging, and 58.3% changed their eating or cooking habits due to the concern. In addition, 79.8% were willing to pay extra for antimicrobial-free food products. Information sources from professionals and authorities were considered more accurate than those from media, the internet, word of mouth, and others.

**Conclusions:**

Chinese consumers had insufficient knowledge and neutral attitudes about AMU in farming production and food regulations in China. A large proportion of the participants were willing to purchase antimicrobial-free food products. Most of them obtained related information from the media. This study highlighted the importance of updated education and effective communication with consumers in China. It helps to develop the reliable foodborne AMR surveillance system along food chain and improve government communication and consumer awareness.

## Introduction

The emergence and spread of food pathogens resistant to antimicrobial drugs, especially those multi- and pan-drug resistant bacteria also known as “superbugs” has created alarming concerns over public health due to significant economic and health impacts (1). AMR is the resistance of a microorganism to an antimicrobial agents that it was previously sensitive to, resulting in medicines ineffective (2). AMR can be induced by overuse and misuse of antimicrobials in human medicine and agriculture or food animal production (3). AMR in human also develops due to the antibiotic residues in animal-derived foods as well as the transmission of resistant bacteria or genes from animals and the environment (4).

Since the 1940s, veterinarians and animal scientists have demonstrated that adding small amounts of antimicrobials (<200 g/ton feed) to feed or water could promote animal growth and higher feed conversion rates, reduce production diseases and improve food animal welfare, which had created a livestock production standard that has resulted in lower production costs and lower meat prices (5). In 2013, the global consumption of antimicrobials used in food-producing animals was around 13,000 tons per year for therapy, prophylaxis, and growth promotion (6), and are expected to be 104,079 tons by 2030 (7). It was equivalent to an estimated use of 100 milligrams of antimicrobials in the livestock for food production of every kilogram of meat for human consumption (8). Previous studies suggested that antimicrobials given in feed for livestock has a significant impact on human health (9, 10).

Association between interventions to reduce AMU in farm animals and decrease in the level of antimicrobial-resistant bacteria in livestock was identified (11). Owing to its importance in the food industry and the potential health risks to the public, AMU in food production has attracted a great deal of societal attention which led to government policy adjustments in recent years (12, 13). To curtail food risk in the spread of AMR via human consumption, it is critical to restrict the presence of AMR bacteria in livestock and animal-derived foods processing by regulations (14). Since 2006, countries such as European Union (EU) members, the United States, Australia, South Korea, and China have limited AMU as animal growth promoters. However, there is still a lack of legislative restrictions in many developing and even some developed countries (15, 16). As one of the largest consumer markets of veterinary antimicrobials, China released revised legislation to further ban AMU as growth promoters in 2019 (17). Starting from January 2020, the antimicrobials which have growth-promoting, prophylaxis, and therapy effects are not allowed in the application for growth-promotion but they can still be used for prophylaxis and therapy in China (18).

Consumers, as one of the important stakeholders, play a significant role in the food supply chain (19). It is imperative to take into consideration the views, concerns, and attitude of the public and consumers so that effective regulations can be elicited and developed to improve the monitoring and surveillance of the food production industry (20). Besides, consumer demand drives market needs for food products without AMU. Therefore, understanding concerns, preferences, and intentions of consumers is crucial for the food industry in the production of acceptable and healthy animal food products (21). In addition, investigation of consumers' willingness to pay extra for the quality as well as consumer concerns helps the development of effective foodborne AMR education systems and communication strategies. This will be ultimately beneficial to societal trust and confidence in the food industry (22).

A scientific report across the EU conducted by European Food Safety Authority (EFSA) showed that European consumers were aware of antimicrobials and AMR, however, the level of understanding of AMU in farming production was not high (23). Similarly, only a few Chilean consumers recognized that the antimicrobials had been widely used in animal farming, and how antimicrobial-resistant bacteria and AMR were transferred via food consumption (24). In some European countries and Canada, consumers also knew little about the farming production diseases (25) and how antimicrobials were being used in livestock production for therapy and prophylaxis use (26). Although the majority of consumers were concerned about AMU and AMR, they believed that AMU in livestock was indispensable as long as it was authorized by veterinarians for productivity, animal welfare, and food quality (27). They preferred that it was exclusively used for therapy, but not for prophylaxis, and assumed that treating healthy animals with antimicrobials might be one of the most important factors contributing to AMR (23). In addition, Chilean consumers recognized that the risk of other chemical residues in food might have a negative effect on their health, such as fertilizers and pesticides (24). This concern urged food companies to provide more information to the consumers about the drug residues in the food provided to the human table.

There were some differences in concerns and intentions regarding AMU in food animals from country to country. Most Chilean consumers considered AMU as a potential risk to their health, so they believed that it was a necessary practice to avoid AMU and were more likely to purchase antimicrobial-free products certified by trusted authorities (24). Nevertheless, Dutch consumers did not regard it as an important issue (27). Another survey conducted in the United States indicated that most consumers thought that animal-derived foods with antimicrobial treatment were safe to eat as long as they were authorized by the Food and Drug Administration (FDA). However, about one-third of consumers still would not purchase foods (28) for consumption if antimicrobials were used. The differences among countries may be related to the different levels of restriction and monitoring by governmental regulations as well as the degrees of policy education, consumer's awareness and knowledge.

As few studies focused on the investigation of consumers' knowledge, attitude, and behavior toward AMU in food production, and there were conflicts and gaps of information among previous studies, further research is needed to identify more convincing evidence and potential reasons contributed to this issue, especially after different levels of limitation were enforced due to changes in regulations. In China, as one of the largest consumer markets globally, research was not done on the Chinese consumers' knowledge, attitude, and behavior since China released the updated restriction on AMU in food production in 2019. This study aimed to investigate the knowledge and awareness, perception and attitude of Chinese consumers toward AMU in food production, and to evaluate their behavioral changes, purchase intention, and confidence in information sources of antimicrobial-free food products.

## Methods

### Survey design and data collection

The online survey questionnaire, consisted of seven sections and 28 questions (Supplementary Appendix), was improved and developed from the previous surveys (23, 29). It could be completed in about 10 minutes. The ethical approval for the survey was obtained from Ethics Committee of Zhejiang University School of Public Health, Reference number ZGL202201-4 Supplementary Image. The consent was obtained from each participant after being explained the aims of the study at the beginning of the survey questionnaire.

The survey was piloted in a sample of 15 participants to ensure that it was easy to understand in the Mandarin version and that there were no technical problems in consent instructions, order and category of questions, and response duration. Then the questionnaire was revised and finalized according to the suggestions before the formal data collection.

The online survey was conducted between February 25 and March 8, 2022. The questionnaire was distributed by Tencent Questionnaire and Wechat app as a web link to invite public consumers to participate in the survey. The minimum recommended sample size was 385 participants based on the standard formula for sample size calculation of simple random sampling, with a 5% margin of error, a confidence interval of 95%, and the current population in China. Given potential missing data, sample size was added by 20% extra to reach 481 samples. The survey was completed by 1065 respondents, which met the required number of samples.

### Data analysis

Descriptive statistics included frequencies or percentages of categorical variables, and means ± standard deviations (SD) and 95% confidence interval (CI) of continuous variables. In terms of Shapiro-Wilk test, histogram, Q-Q plot, and the numbers of samples, all the continuous variables were considered as near normal distribution. Levene test was used to determine the homogeneity of variances. If variances showed heterogeneity, *t*-test was replaced by *t*'-test, and one-way analysis of variance (ANOVA) test was replaced by Welch test.

The differences in knowledge and awareness were performed by independent *t*-test when compared with “gender,” and by ANOVA test and Bonferroni *post-hoc* test for other demographic groups. Factor analysis with Direct Oblimin rotation was conducted to identify underlying patterns of the attitudes. Initial eigenvalues were used to screen the number of factors. Kaiser-Meyer-Olkin (KMO) measure and Bartlett's test of Sphericity were used to verify the adequacy of factor analysis. The internal reliability of each scale was then tested to evaluate the contribution of each item to the factor with Cronbach's α coefficient. Spearman correlations between the identified scales were employed, to determine the relationship between the mean scores of attitudes. The differences in attitudes among demographic characteristics were also evaluated. Spearman correlations were employed to determine the relationship between the percentage of the intention to pay extra. The difference in intention was performed by Chi-square test for all demographic groups. Similarly, factor analysis with Direct Oblimin rotation was conducted to identify information sources. The confidence scales of information sources were constructed by computing the mean value of the loading items for each factor. Measure of sampling adequacy and internal reliability test were also performed. The dependence value of Cronbach's α coefficient was defined as acceptable when it was ≥0.70. *P* < 0.05 was considered statistically significant. Data analysis was conducted in IBM SPSS Statistics for Mac version 26 (IBM Corporation, Armonk, NY, USA). GraphPad Prism for Mac version 8.0 (GraphPad Software Inc., San Diego, CA, USA) was used to create charts.

## Results

### Demographic information

Demographic characteristics were presented (Supplementary Table 1), including gender, age, highest education level, work status, occupation, household income, the number of household adults and children, and place of residence. It showed that the majority of the participants were female (62.5%, 666/1,065), at the age of 18 to 40 years (60.7%, 646/1,065), with education degree of bachelor and higher (61.7%, 657/1,065), and employed full-time (57.9%, 617/1,065).

### Knowledge and awareness of AMR and AMU in food production, and the current situation in China

General awareness of AMR or antibiotic resistance was high. Of respondents, 75.0% (799/1,065) claimed that they had heard of antibiotic resistance or AMR, 48.2% (513/1,065) knew what AMR was, 18.4% (196/1,065) felt that they did not know it, while 33.4% (356/1,065) were not sure about the definition of AMR.

**Figure 1:**
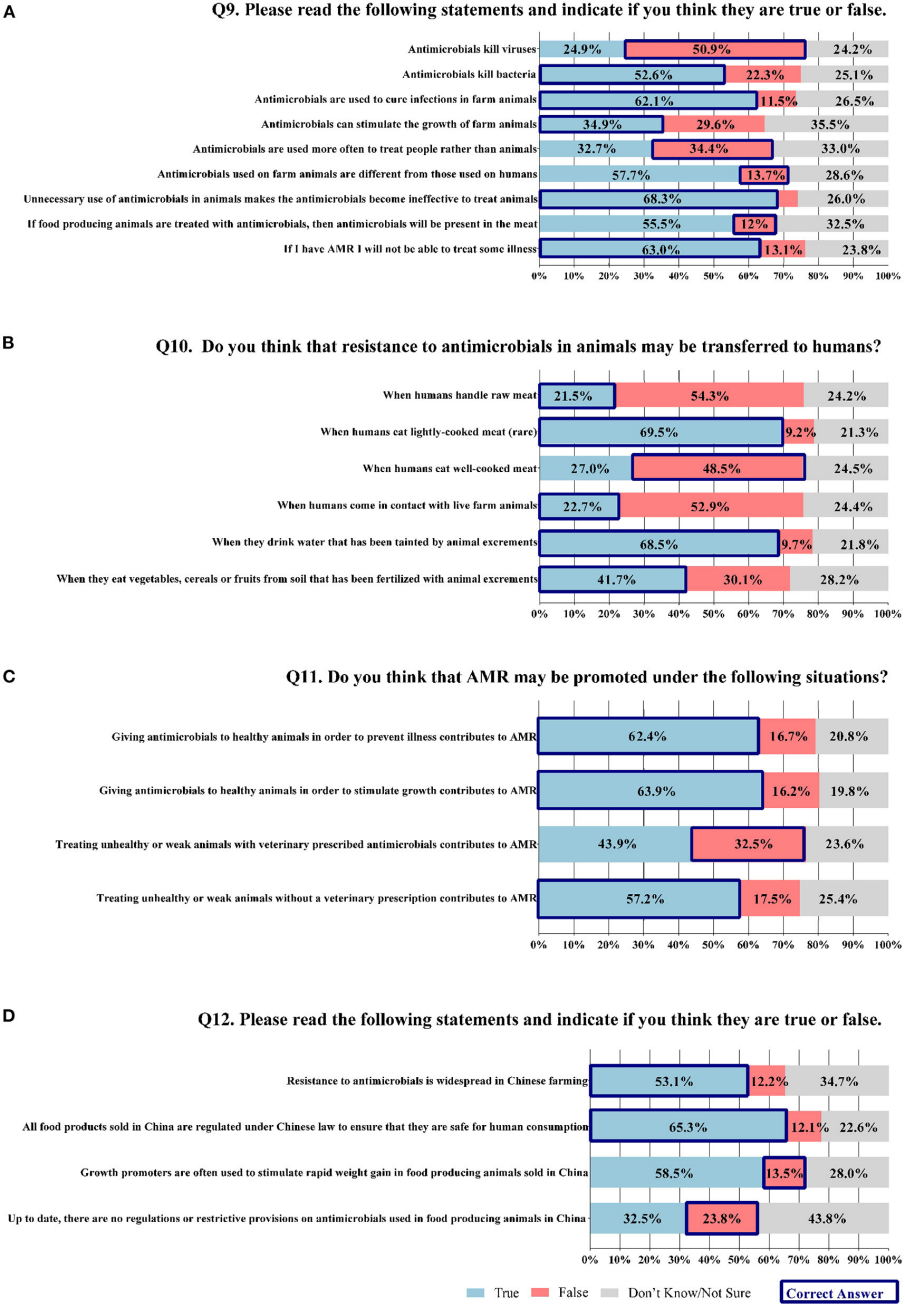
Consumers' knowledge and awareness of AMR and AMU in food production. (N=1065). **(A)** Q9: 9 questions investigating general knowledge of AMR and AMU in food production. **(B)** Q10: 6 questions assessing knowledge of how resistant bacteria transferred from animals to humans. **(C)** Q11: 4 questions measuring knowledge of how AMR may be promoted. **(D)** Q12: 4 questions evaluating knowledge and awareness of current situation in China.

However, the knowledge and awareness of AMR and AMU in food production was not high. Respondents gave 8.8±3.7 (95%CI 8.6–9.0) correct answers for 18 statements from Q9 to Q11, which meant that participants were able to reply correctly with 48.9% (8.8/18) on average to these statements (Figures 1A,C). For the general knowledge in Q9, the respondents gave 3.9 ± 2.0 (95%CI 3.8–4.0) correct answers for nine statements, i.e., 43.5% (3.9/9) on average of these statements. Most respondents correctly answered that antimicrobial agents could kill or inhibit the growth of bacteria (52.6%, 560/1,065) but not viruses (50.9%, 542/1,065). It was used to cure infections or preventing diseases in animals (62.1%, 661/1,065). A large majority of participants were aware that unnecessary use of the antimicrobials in animals made antimicrobials ineffective in the needed treatment of animal diseases (68.3%, 727/1,065), and humans will not be able to be cured if having AMR foodborne pathogens infected (63.0%, 671/1,065). Whereas, only a few respondents were aware that the antimicrobial could stimulate the growth of animals (34.9%, 372/1,065 assessed correctly); the antimicrobials were more often used to treat animals than humans (34.4%, 366/1,065 assessed correctly); the antimicrobials used on farm animals were usually the same with those used on humans (13.7%, 146/1,065 assessed correctly); if food-producing animals were treated with antimicrobials, antimicrobials were not always present in the meat, depending on the dosage (12.0%, 128/1,065 assessed correctly). Besides, participants were not highly aware of how resistant bacteria could be transferred from animals to humans in Q10. They gave 2.7±1.6 (95%CI 2.6–2.8) correct answers for 6 statements, i.e., 45.4% (2.7/6) on average of these statements. Most respondents correctly answered that antimicrobial-resistant bacteria could be transferred when eating rare or lightly cooked meat (69.5%, 740/1,065), as well as when drinking water tainted by animal excrement (68.5%, 730/1,065). Nearly half (41.7%, 444/1,065) of respondents were aware that antimicrobial-resistant bacteria could be transferred through consumption of food from soil that was fertilized with animal excrement. Nevertheless, they underestimated the risk of transfer from handling raw meat (21.5%, 229/1,065 replied correctly) and from contacting live farm animals (22.7%, 242/1,065 replied correctly). They were overly worried about the risk of eating well-cooked meat (48.5%, 517/1,065 replied correctly). In addition, as for the contribution of AMR in Q11, respondents gave 2.2 ± 1.3 (95%CI 2.1–2.2) correct answers for four statements, i.e., 54.0% (2.2/4) on average was correct for these statements. The majority of respondents knew that AMR could be induced by giving antimicrobials to healthy animals for disease prevention (62.4%, 665/1,065) and growth stimulation (63.9%, 681/1,065), and by treating unhealthy or weak animals without veterinary prescribed antimicrobials (57.2%, 609/1,065). While only 32.5% (346/1,065) correctly answered that treating sick animals with veterinary prescribed antimicrobials would not contribute to AMR under normal situations.

It was discovered that there was a significant difference in the knowledge between the education levels (F = 16.3, *P* < 0.001) (Supplementary Table 2). Those who had completed higher education, i.e., bachelor, postgraduate and higher degrees, were significantly more knowledgeable about AMR and AMU in food production, compared to other groups of respondents with junior high school or lower education (both *P* < 0.001), senior high school (both *P* < 0.001), and college (both *P* < 0.001). Participants in the “postgraduate and higher degrees” group were also significantly more knowledgeable than those who were bachelors (*P* = 0.044). When compared with work status, those who were “employed full-time (≥30 h per week)” had significantly higher awareness than those who were “unemployed” (*P*= 0.008). In addition, there was a significant difference among multiple income groups. Those “prefer not to say” had lower knowledge level than respondents whose total income was “¥ 500,000–¥ 1,000,000 per annum” (*P* = 0.014). Those with total household income “¥ 200,000–¥ 500,000 per annum” had higher awareness of AMR and AMU in food production, compared to respondents with household income “under ¥ 50,000 per annum” (*P* < 0.001), and “¥ 50,000–¥ 100,000 per annum” group (*P* = 0.007). There was no statistically significant difference in comparisons among “gender,” “age,” “household adults” and “household children” (all *P* > 0.05).

Knowledge and awareness of current situations of AMU in food production in China were also low. Respondents gave 1.6±1.0 (95%CI 1.5–1.6) correct answers for 4 statements, which meant that participants were able to answer correctly to 38.9% (1.6/4) on average of these statements (Figure 1D). Over half of the respondents were aware that AMR was widespread in Chinese farming (53.1%, 565/1,065), and all food products sold in China were regulated under Chinese law to ensure that they were safe for human consumption (65.3%, 695/1,065). Most of them had little knowledge of the current provisions on the restriction of antimicrobials used in farming and growth promotion (23.8%, 253/1,065, and 13.5%, 144/1,065 replied correctly). In addition, it was found that the awareness of AMU in China was significantly influenced by work status (*F* = 3.2, *P* = 0.007). The respondents who were employed full-time gave 1.6 ± 1.0 (95%CI 1.6-1.7) correct answers which was significantly higher than those who were retired, 1.2 ± 0.9 (95%CI 1.0-1.4), (*P* = 0.011). There was no significant difference among different groups of “gender,” “age,” “highest level of education,” “household income”, “household adults,” and “household children” (all *P* > 0.05).

### Perception and attitude toward AMR and AMU in food production, and the current situation in China

**Figure 2:**
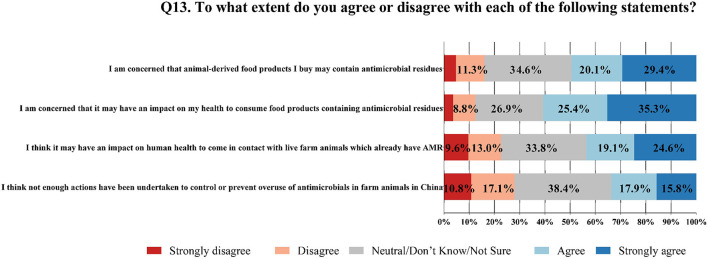
Consumers' perceptions and attitudes towards AMR and AMU in food production, and the current situation of that in China (N = 1065).

The percentage scores of participants for each statement were reported (Figure 2). Results revealed that 49.5% (527/1,065) of them were concerned that animal-derived food products they bought might contain antimicrobial residues, while 60.7% (646/1,065) thought that it might have an adverse impact on their health after consumption of food products containing antimicrobial residues. In addition, 43.7% (465/1,065) were worried that it might have an impact on human health after contact with live farm animals which already had AMR. As to the supervision and guidance measures in Chinese farming industry, 33.7% (359/1,065) of respondents considered that policies and actions taken to control or prevent the overuse of antimicrobials in food-producing animals were not sufficient.

The exploratory factor analysis of perception and attitude was yielded with a two-factor solution which explained 82.3% of the variance. Factor one consisted of three concern statements related to AMR and AMU in food production, which was renamed the scale of antimicrobial-use attitude. Factor two included one perception related to current situations of that in China, which was renamed the scale of China-situation attitude (Table 1). The internal consistency for factor one was estimated using Cronbach's α coefficient, which was α = 0.84. Scale scores and factor correlations (Mean ± SD, 95% CI) were presented (Table 2). There was a significant negative correlation between the two factors (r = −0.220, *P* < 0.01), meaning that the participants who were more concerned about the issue were less likely to perceive that insufficient measures were being taken to limit the overuse of antimicrobials.

**Table 1:** The pattern matrix of exploratory factor analysis of perceptions and attitudes.

**Statements of perceptions and attitudes**	**Factor 1**	**Factor 2**
I am concerned that animal-derived food products I buy may contain antimicrobial residues	0.921	
I am concerned that it may have an impact on my health to consume food products containing antimicrobial residues	0.912	
I think it may have an impact on human health to come in contact with live farm animals which already have AMR	0.743	
I think not enough actions have been undertaken to control or prevent the overuse of antimicrobials in farm animals in China		0.982

Principal component analysis was used as the extraction method and Oblimin with Kaiser Normalization as the rotation method. Factor loadings <0.30 were suppressed and not presented in the table. KMO measure of sampling adequacy = 0.679, Bartlett's test of Sphericity *P*-value <0.001.

**Table 2:** Spearman correlation between knowledge, attitudes, and intentions.

**Scale**	**Mean ±SD (95%CI)**	**Spearman correlation r**_**s**_				
	**Or N (%)**	**^a^**	**^b^**	**^c^**	**^d^**	**^e^**
Antimicrobial-use knowledge^a^	8.8 ± 3.7(8.6–9.0)	1				
China-situation knowledge^b^	1.6 ± 1.0(1.5–1.6)	0.364^**^	1			
Antimicrobial-use attitude^c^	3.6 ± 1.0(3.5–3.6)	0.153^**^	0.061^*^	1		
China-situation attitude^d^	2.9 ± 1.2(2.8–3.0)	0.033	−0.103^**^	−0.220^**^	1	
Willingness to pay^e^	Yes: 850 (79.8%)	−0.166^**^	−0.125^*^	0.142^**^	0.049	1

a Sum score of the answers from Q9 to Q11 (0 = don't know/not sure, 0 = false answer, 1 = correct answer).

b Sum score of the answers in Q12 (0 = don't know/not sure, 0 = false answer, 1 = correct answer).

c Mean score of the first three statements in Q13 (1 = strongly disagree, 2=disagree, 3 = neutral/don't know/not sure, 4=agree, 5=strongly agree).

d Mean score of the fourth statement in Q13 (1 = strongly disagree, 2 = disagree, 3 = neutral/don't know/not sure, 4=agree, 5=strongly agree).

e Rank answer of Q15 respondence (1 = yes, 2 = no).

* *P* < 0.05,

** *P* < 0.01.

In addition, it was found that the scales of antimicrobial-use attitude and China-situation attitude were affected by age (*F* = 9.2, *P* < 0.001) (Supplementary Table 3). The middle-aged respondents aged 31–40, 41–50, and 51–60 were more worried about AMU in food production than the younger respondents who were 18–30 years old (*P* < 0.001, *P* < 0.001, *P* = 0.038). Similarly, the younger respondents who were 18–30 years old perceived to a less extent that insufficient measures were being taken to control the overuse of antimicrobials, compared to those aged 41-50 (*P* < 0.001), and 51–60 (*P* < 0.001). Differences could also be observed among the household income groups both in the scales of antimicrobial-use attitude and China-situation attitude. For the scale of antimicrobial-use attitude, the respondents whose household income was “¥ 200,000–¥ 500,000 per annum” replied with higher concern than those with “under ¥ 50,000 per annum” (*P* = 0.026) and “¥ 100,000–¥ 200,000 per annum” (*P* = 0.033). For the scale of China-situation attitude, the respondents whose household income was “¥ 50,000–¥ 100,000 per annum” perceived to a less extent that insufficient measures were being taken to control the overuse of antimicrobials than the groups with household income at “¥ 200,000–¥ 500,000 per annum” (*P* = 0.003) and “¥ 500,000–¥ 1,000,000 per annum” (*P* = 0.015). Similarly, the respondents whose household income was “not stable/not sure” perceived to a less degree that insufficient measures were being taken to control the overuse of antimicrobials than the groups “¥ 200,000–¥ 500,000 per annum” (*P* = 0.027) and “¥ 500,000–¥ 1,000,000 per annum” (*P* = 0.019). When compared with the work status, the participants who were students were less worried about AMU in food production than those who were employed full-time (*P* = 0.005). Likewise, students perceived less that insufficient measures were taken to control the overuse of antimicrobials than those who were retired (*P* = 0.028). When compared with the highest education level, the respondents who had completed postgraduate and higher degrees perceived to a higher extent that insufficient measures were taken to control the overuse of antimicrobials, compared to others who completed the education of senior high school (*P* = 0.002), college (*P* < 0.001), and bachelor (*P* = 0.001). However, there was no significant difference in comparison among “gender,” “household adults,” and “household children” for both the scales (all *P* > 0.05).

### Behavioral changes due to the concern of AMR and AMU in farm animals

**Figure 3:**
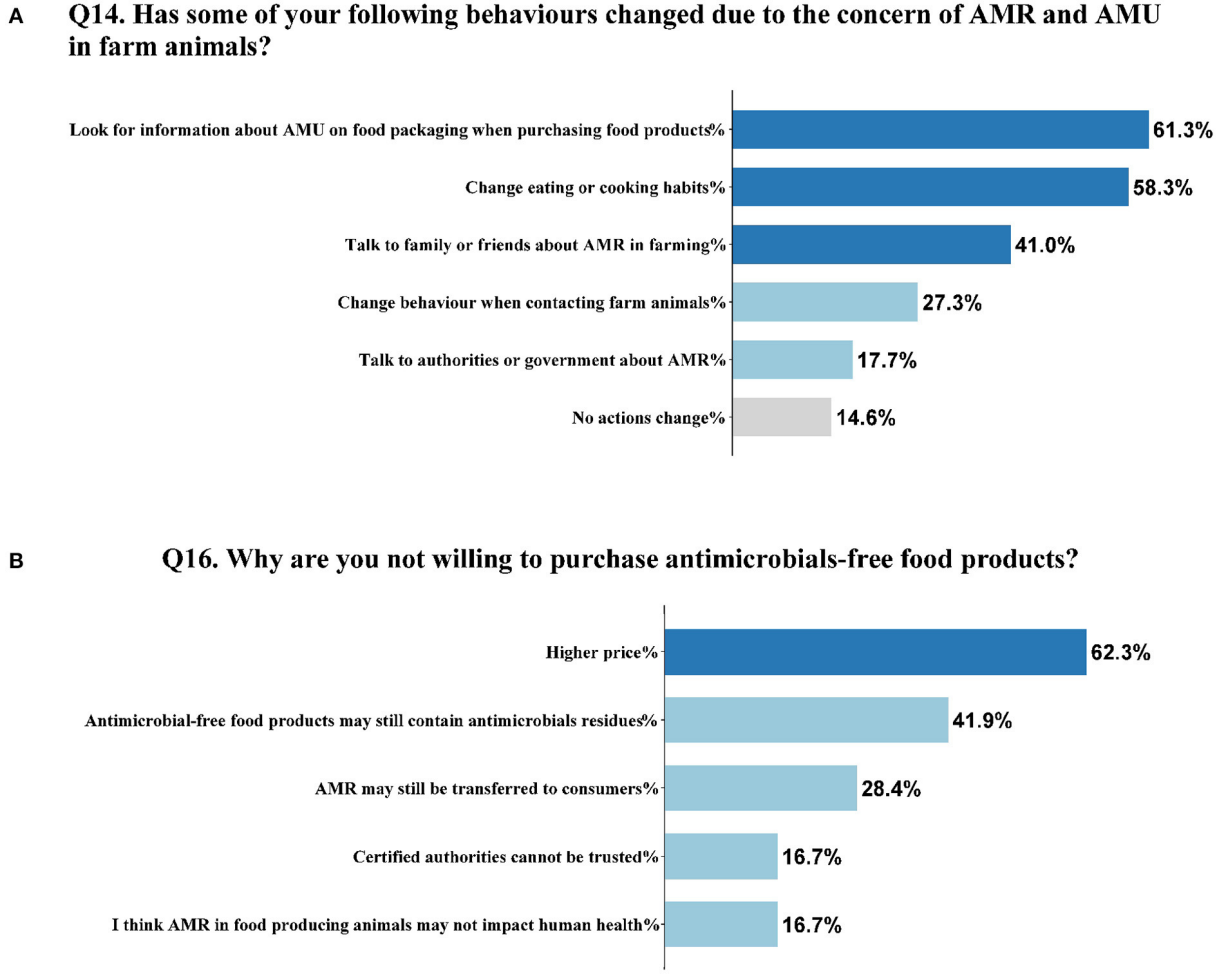
Behavioral changes, and the reasons why consumers were not willing to pay extra for antimicrobials-free food products. **(A)** Behavioral changes due to the concern of AMR and AMU in farm animals (N = 1065). **(B)** The reasons why consumers were not willing to pay extra for antimicrobials-free products (N =215).

Of the participants, 61.3% (653/1,065) claimed that they were more likely to look for specific information about AMU on food packaging when they purchased food products, and 58.3% (621/1,065) changed their eating or cooking habits due to the concern of AMR and AMU in farming (Figure 3A). In addition, 41.0% (437/1,065) discussed AMR with their families or friends more often, 27.3% (291/1,065) changed their behavior such as using enhanced protective measures when contacting live food-producing animals, and 17.7% (189/1,065) talked to authorities or government about AMR, while 14.6% (155/1,065) answered that no actions were taken.

### Willingness and intention to pay extra for antimicrobial-free food products

A large proportion (79.8%, 850/1,065) of the respondents were willing to pay a higher price for antimicrobial-free food products, whereas 20.2% (215/1,065) were not willing to pay extra. Most respondents viewed AMR and AMU as potential risks to their health, as the Spearman correlation showed a statistical significance in difference between the attitude and the willingness to pay (*r* = 0.142, *P* < 0.01) (Table 2). Among these 215 respondents, 62.3% (134/215) stated that the higher price was one of the barriers to purchasing antimicrobial-free products, and 41.9% (90/215) claimed that they were still concerned about the antimicrobial residues in the products (Figure 3B). Of 215 participants, 28.4% (61/215) thought that AMR might still be transferred to the public, and 16.7% (36/215) considered that the certified authorities could not be trusted. Nevertheless, only 16.7% (36/215) did not regard AMR as a potential risk to health.

It was discovered that there were significant differences in the willingness to pay compared among age (χ^2^ = 32.9, *P* < 0.001), work status (χ^2^ = 21.8, *P* < 0.001), and household income (χ^2^ = 40.9, *P* < 0.001) (Supplementary Table 4). For age groups, 86.1% (155/180) of 31–40 years old (*P* = 0.001), 89.0% (97/109) of 41–50 years old (*P* = 0.001), and 92.5% (74/80) of 51–60 years old (*P* < 0.001), were willing to pay higher prices for antimicrobial-free food products, all significantly >74.6% (482/646) of participants who were 18–30 years old willing to pay a higher price. When compared with the work status, 83.1% (513/617) of the full-time employed respondents showed the willingness to pay more for antimicrobial-free food products, while 71.1% (189/266) of the students showed willingness (*P* < 0.001). When compared with household income, 91.0% (152/167) of the subjects in the “¥ 200,000–¥ 500,000 per annum” group showed the willingness to pay more for antimicrobial-free food products, significantly >67.9% of the participants in the “under ¥ 50,000 per annum” group (*P* < 0.001), 79.4% (224/282) in the “under ¥ 50,000 per annum” group (*P* = 0.001), 73.3% (33/45) in the “Not stable/Not sure” group (*P* = 0.002), and 67.0% (63/94) in the “Prefer not to say” group (*P* < 0.001). Of the subjects, 84.0% (226/269) in the “¥ 100,000–¥ 200,000 per annum” group answered they were willing to pay, significantly more than 67.9% (93/137) in the “under ¥ 50,000 per annum” group and 67.0% (63/94) in the “Prefer not to say” group (both *P* < 0.001). However, there was no significant difference compared among “gender,” “highest level of education,” “household adults,” and “household children” (all *P* > 0.05).

### Communication and confidence in sources

**Figure 4:**
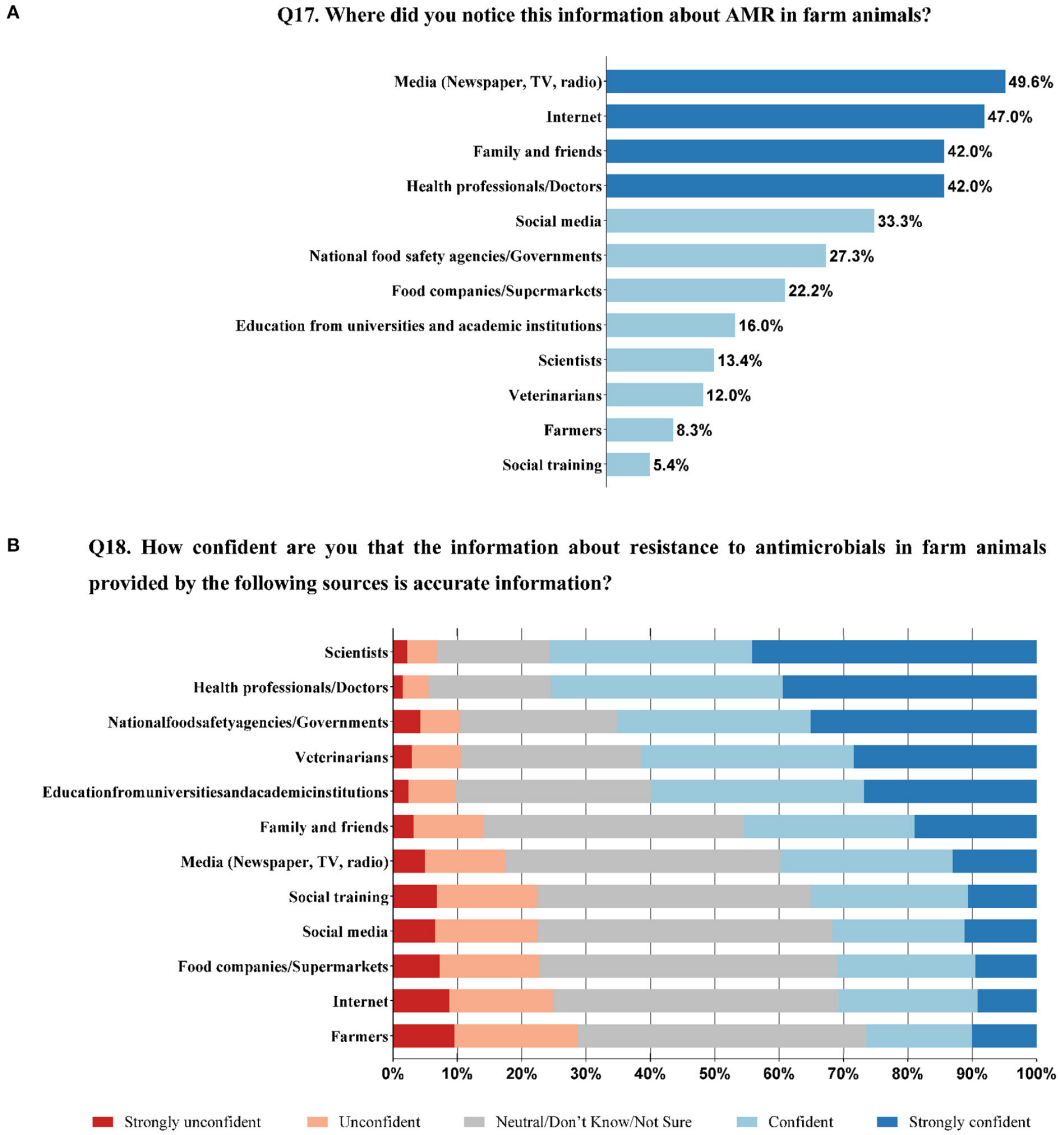
Information sources about AMR and AMU in farming **(A)**, and consumers' confidence in information sources **(B)** (N = 1065).

The percentage score of participants for each information source were reported (Figure 4A). Most respondents obtained the information on AMR in farm animals from the media (49.6%, 528/1,065), followed by the Internet (47.0%, 501/1,065) and family and friends (42.0%, 447/1,065).

The exploratory factor analysis of information sources yielded a two-factor solution which explained 58.2% of the variance. Factor one consisted of seven sources, i.e., social media, Internet, media (newspaper, TV, radio), farmers, social training, food companies/supermarkets, family and friends, which was renamed as the scale of media, Internet, word of mouth, others. And factor two included five sources, i.e., scientists, health professionals/doctors, veterinarians, education from universities and academic institutions, and the national food safety agencies/governments, which was renamed as the scale of professionals and authorities. The internal consistency for each factor was assessed with Cronbach's α coefficient, whose α were 0.86 and 0.84, respectively for factor 1 and factor 2 (Table 3).

**Table 3:** The pattern matrix of exploratory factor analysis of information sources.

**Information sources**	**Factor 1**	**Factor 2**
Social media	0.865	0.144
Internet	0.832	0.044
Media (Newspaper, TV, radio)	0.794	0.246
Farmers	0.678	0.286
Social training	0.607	0.323
Food companies/Supermarkets	0.588	0.152
Family and friends	0.508	0.300
Scientists	0.082	0.828
Health professionals/Doctors	0.154	0.822
Veterinarians	0.208	0.723
Education from universities and academic institutions	0.298	0.687
National food safety agencies/Governments	0.346	0.673

Principal component analysis was used as the extraction method and Oblimin with Kaiser Normalization as the rotation method. KMO measure of sampling adequacy = 0.889, Bartlett's test of Sphericity *P*-value <0.001.

Not all sources were considered accurate information about AMR and AMU in food production. Mean±SD (95%CI) scale scores and factor correlations were presented (Table 4). Five out of twelve sources were deemed to be more accurate, while the other seven sources were perceived as being less accurate (Figure 4B) (Supplementary Table 5). In other words, sources from professionals and authorities were considered more accurate than that from media, the Internet, word of mouth, and others. However, there was a significant positive correlation between the two scales (*r* = 0.494, *P* < 0.01), indicating that those who were confident in the professional sources were also confident in the media sources and vice versa. The top three trusted sources were scientists (75.7%, 806/1,065 confident/strongly confident), health professionals/doctors (75.5%, 804/1,065), and national food safety agencies/governments (65.3%, 695/1,065), while the most untrusted sources were farmers (26.5%, 282/1,065 strongly unconfident/unconfident). However, 28.9% (308/1,065) of the respondents answered neutral/don't know/not sure about media, Internet, word of mouth, others, and it might be due to the lack of knowledge and awareness of this issue.

**Table 4:** Descriptive statistics (mean, standard deviation, and correlation coefficient) for each information source scale.

**Scale**	**Mean ±SD (95%CI)**	**Spearman correlation r**_**s**_	
		**^a^**	**^b^**
**Media, Internet, word of mouth, others** ^a^	3.2 ± 0.8 (3.1–3.2)	1	
**Professionals and authorities** ^b^	3.9 ± 0.8 (3.9–4.0)	0.494^**^	1

a Mean score of the seven sources, i.e., social media, Internet, media (newspaper, TV, radio), farmers, social training, food companies/supermarkets, family and friends (1 = strongly unconfident, 2 = unconfident, 3 = neutral/don't know/not sure, 4 = confident, 5 = strongly confident).

b Mean score of the five sources, i.e., scientists, health professionals/doctors, veterinarians, education from universities and academic institutions, national food safety agencies/governments (1 = strongly disagree, 2 = disagree, 3 = neutral/don't know/not sure, 4 = agree, 5 = strongly agree).

** *P* **<** 0.01.

## Discussion

In this study, consumers were clearly aware of AMR, however, the knowledge of AMU in farming production was not high, which matched the results in the surveys conducted in Europe and Chile (24). We also found that those who were employed full-time, who had completed higher education, and earned higher income, were more likely to be knowledgeable about AMR and AMU in food production. Similar results were published in a previous survey in EU countries (23). In addition, the participants were observed to have insufficient awareness in terms of the related food regulations in China. Although China has taken representative actions to minimize and contain foodborne AMR, e.g., released a national action plan in 2016 (30) and drafted several international regulations (31, 32), our study indicated that it was necessary for the authorized institutions to educate the public, especially those with low levels of education, to improve their awareness and understanding of AMU in food production and the government policy measures limiting the use of antimicrobials.

Previous studies indicated that there were some differences in general concern about AMU in livestock animals as a potential risk to public health from country to country. For instance, most Chilean consumers considered AMU as a potential risk to their health (24), while Dutch consumers did not regard it as an important issue (27). It may be related to the policy regulations and the extent of restrictions enforced by the government. In Netherlands AMU in farm production has been very limited, while Chile still lacks restrictions on this issue. In this study, almost half of the consumers viewed the issues of antimicrobial residues in food products and AMR from live farm animals in China as potential risks to human health. It was found that the middle-aged respondents were more worried about AMU in food production. This population also perceived more that insufficient measures were taken to control the overuse of antimicrobials compared to the young respondents. The respondents with higher income replied with higher concern and perceived more that insufficient measures were taken to control the overuse of antimicrobials than those with lower income. In addition, it suggested that the participants, who were less knowledgeable about AMR and food regulations, were less concerned and were less likely to perceive that insufficient measures were taken to limit the overuse of antimicrobials. As China released and enforced legislation limiting AMU only 2 years ago, it may explain insufficient awareness among the public and why they still hold high concerns toward current actions and measures of Chinese government.

In terms of the behavioral changes of consumers, it was discovered in this study that more than half of the participants claimed that they were more likely to look for specific information about AMU on food packaging when they purchased food products. They changed their eating or cooking habits due to the concern of AMR and AMU in farming. This should urge food companies to provide detailed information to the consumers about the antimicrobials and chemicals used during production and certify that drug residues are within policy and regulation limit.

It was identified in previous studies that consumers in the US, Canada, Germany, and Chile were willing to pay a higher price for food products free of antimicrobials, depending on their knowledge and attitude (24, 26, 33). We found that a large proportion of the respondents in China were willing to pay more for antimicrobial-free food products as well. It might be related to the facts that most respondents viewed AMR and AMU as potential risk to their health based on Spearman correlation. On the other hand, only a small part of the respondents were not willing to pay extra. This was mainly due to the higher price and ignorance of the possibility of antimicrobial residues in the products.

In terms of the research on risk communication from the perspective of authorities and food companies, there are three essential and necessary components when communicating information with consumers (34). First, communication must be rapid and accurate, which helps the public to understand well-about a newly released policy or changed regulations. Consumer research suggested that the public was delighted to be educated thoroughly and completely about food safety issues (24). Second, the administrative personnel of authority should be credible and reliable who makes the announcement. They must be independent without any interest in or relationship with the food companies or suppliers. This should increase the trust of consumers. Third, as for food companies, it is essential to evaluate the scientific correctness and effectiveness of food labels with timely updates. The majority of consumers have far less trust in the food industry and the media as reliable sources of information (23). They tended to have higher confidence in the veterinarians, scientists, and government authorities. They also believed that it was critical to get access to clear and reliable information from food providers which should display important information and health concerns on food labels. However, further studies are needed on whether consumer behavior would change even if the food label information is clear and effective (35).

With the aspect of communication and information sources, most respondents obtained information on AMR in farm animals from the media, followed by the Internet and family and friends in this survey although sources from professionals and authorities were considered more accurate. This result was similar to a previous report that the majority of consumers preferred scientists and food safety agencies as the more reliable sources of information compared to the food industry and the media (23). However, it indicated that those who were less confident in the professional sources were also less confident in the media sources and vice versa. It might be related to the lack of knowledge and awareness of this issue resulting in the overall low level of confidence.

This study was the first to investigate the knowledge, and attitudes of Chinese consumers toward AMU in food production, and evaluated their behavioral changes, purchase intention, and confidence in information sources of antimicrobial-free food products. However, it also had limitations. Given the survey method based on an online questionnaire, only participants with access to technology equipment were reached. Furthermore, the samples were imbalanced, e.g., most participants were 18 to 30 years old (60.7%), and 31.5% of respondents were from Zhejiang Province, therefore the answers may be geographically and demographically biased because the participants were not enough to be representative of Chinese consumers as a whole. Nonetheless, this study provides a valuable contribution to the exploratory investigation of knowledge, attitude, and behavior of Chinese consumers. Further research is needed to widen population representation.

## Conclusions

This study demonstrated that most Chinese consumers were aware of AMR, however, the knowledge of AMU in farming production was not high. They were observed to have insufficient awareness in terms of the related food regulations in China as well. Almost half of the participants viewed the issues of antimicrobial residues in food products and AMR from live farm animals as potential risks to human health. More than half of the respondents claimed that they had been more likely to look for specific information about AMU on food packaging when they purchased food products, and had changed their eating or cooking habits due to the concern of AMR and AMU in farming. A large proportion of the participants were willing to pay a higher price for antimicrobial-free food products. Most of them obtained information on AMR and AMU in farm animals from the media, whereas information sources from professionals and authorities were considered more accurate and reliable than those from media, the Internet, word of mouth, and others.

The identified evidence in this research highlighted the importance of updated education for the public and effective communication with the consumers, which could help improve foodborne AMR surveillance system along food chain and communication strategies. However, this study was limited to a biased younger population in a more developed province, Zhejiang, in China. Further studies should expand to other populations and regions on AMR and AMU in food production.

## Data availability statement

The raw data supporting the conclusions of this article will be made available by the authors, without undue reservation.

## Ethics statement

The studies involving human participants were reviewed and approved by Ethics Committee of Zhejiang University School of Public Health. The patients/participants provided their informed consent to participate in this study.

## Author contributions

Study design: QD, XD, YZ, and MY. Collection of data: QD, JG, YZ, and MY. Analysis and interpretation of data: QD, DH, YZ, and MY. Drafting the manuscript: QD, YZ, and MY. All authors have read, revised, and approved the final version of the manuscript.

## Funding

This research was financially supported by the Fundamental Research Fund for the Zhejiang Provincial Universities (grant number 2021XZZX029), the Key Laboratory of Intelligent Preventive Medicine of Zhejiang Province (grant number 2020E10004), the Leading Innovative and Entrepreneur Team Introduction Program of Zhejiang (grant number 2019R01007), and the Key Research and Development Program of Zhejiang Province (grant number 2020C03002) China.

## Conflict of interest

The authors declare that the research was conducted in the absence of any commercial or financial relationships that could be construed as a potential conflict of interest.

## Publisher's note

All claims expressed in this article are solely those of the authors and do not necessarily represent those of their affiliated organizations, or those of the publisher, the editors and the reviewers. Any product that may be evaluated in this article, or claim that may be made by its manufacturer, is not guaranteed or endorsed by the publisher.

## References

[1] Howard SJ Catchpole M Watson J Davies SC. Antibiotic resistance: global response needed. Lancet Infect Dis. (2013) 13:1001–3. 10.1016/S1473-3099(13)70195-6 24252476

[2] World Health Organization. Antimicrobial resistance. (2021). Available online at: https://www.who.int/news-room/fact-sheets/detail/antimicrobial-resistance (accessed January 12, 2022).

[3] World Health Organization. Global antimicrobial resistance surveillance system: manual for early implementation. Geneva: World Health Organization (2015). 36 p. Available online at: https://apps.who.int/iris/handle/10665/188783 (accessed December 31, 2021).

[4] Van den Bogaard AE Stobberingh EE. Epidemiology of resistance to antibiotics: links between animals and humans. Int J Antimicrob Agents. (2000) 14:327–35. 10.1016/S0924-8579(00)00145-X 10794955

[5] Ricke SC Jarquin R Hanning I. 16 - Antimicrobials in animal feed: benefits and limitations. In: Fink-Gremmels J editor. Animal Feed Contamination. Woodhead Publishing Series in Food Science, Technology and Nutrition. Woodhead Publishing (2012). p. 411–431 10.1533/9780857093615.4.411

[6] Van Boeckel TP Glennon EE Chen D Gilbert M Robinson TP Grenfell BT. Reducing antimicrobial use in food animals. Science. (2017) 357:1350–2. 10.1126/science.aao1495 28963240 PMC6510296

[7] Tiseo K Huber L Gilbert M Robinson TP Van Boeckel TP. Global trends in antimicrobial use in food animals from 2017 to 2030. Antibiotics. (2020) 9:E918. 10.3390/antibiotics9120918 33348801 PMC7766021

[8] Walpole SC Prieto-Merino D Edwards P Cleland J Stevens G Roberts I. The weight of nations: an estimation of adult human biomass. BMC Public Health. (2012) 12:439. 10.1186/1471-2458-12-439 22709383 PMC3408371

[9] Van Boeckel TP Brower C Gilbert M Grenfell BT Levin SA Robinson TP. Global trends in antimicrobial use in food animals. Proc Natl Acad Sci USA. (2015) 112:5649–54. 10.1073/pnas.1503141112 25792457 PMC4426470

[10] Aarestrup FM. The livestock reservoir for antimicrobial resistance: a personal view on changing patterns of risks, effects of interventions and the way forward. Philos Trans R Soc Lond B Biol Sci. (2015) 370:20140085. 10.1098/rstb.2014.0085 25918442 PMC4424434

[11] Tang KL Caffrey NP Nóbrega DB Cork SC Ronksley PE Barkema HW. Restricting the use of antibiotics in food-producing animals and its associations with antibiotic resistance in food-producing animals and human beings: a systematic review and meta-analysis. The Lancet Planetary Health. (2017) 1:e316–27. 10.1016/S2542-5196(17)30141-9 29387833 PMC5785333

[12] Ben Y Fu C Hu M Liu L Wong MH Zheng C. Human health risk assessment of antibiotic resistance associated with antibiotic residues in the environment: a review. Environ Res. (2019) 169:483–93. 10.1016/j.envres.2018.11.040 30530088

[13] Bacanli M Başaran N. Importance of antibiotic residues in animal food. Food and Chemical Toxicology. (2019) 125:462–6. 10.1016/j.fct.2019.01.033 30710599

[14] Gonzalez Ronquillo M Angeles Hernandez JC. Antibiotic and synthetic growth promoters in animal diets: Review of impact and analytical methods. Food Control. (2017) 72:255–67. 10.1016/j.foodcont.2016.03.001

[15] Salim HM Huque KS Kamaruddin KM Beg MDAH. Global restriction of using antibiotic growth promoters and alternative strategies in poultry production. Sci Prog. (2018) 101:52–75. 10.3184/003685018X15173975498947 29467062 PMC10365203

[16] World Health Organization, Food and Agriculture Organization of the United Nations, World Organization for Animal Health. Monitoring Global Progress on Antimicrobial Resistance: Tripartite AMR Country Self-Assessment Survey (TrACSS) 2019-2020: Global Analysis Report. World Health Organization (2021). Available online at: https://apps.who.int/iris/handle/10665/340236 (accessed January 10, 2022).

[17] Ministry of Agriculture and Rural Affairs of the People's Republic of China. Ministry of Agriculture and Rural Affairs Announcement No. 194. (2019). Available online at: http://www.xmsyj.moa.gov.cn/zcjd/201907/t20190710_6320678.htm (accessed January 10, 2022).

[18] Lim MSM Grohn YT. Comparison of China's and the European Union's Approaches to Antimicrobial Stewardship in the Pork Industry. Foodborne Pathog Dis. (2021) 18:567–73. 10.1089/fpd.2020.2887 33794668

[19] Kamrath C Wesana J Bröring S De Steur H. What do we know about chain actors' evaluation of new food technologies? A systematic review of consumer and farmer studies. Compr Rev Food Sci Food Saf. (2019) 18:798–816. 10.1111/1541-4337.12442 33336924

[20] Bennett RM Anderson J Blaney RJP. Moral intensity and willingness to pay concerning farm animal welfare issues and the implications for agricultural policy. J Agricult Environ Ethics. (2002) 15:187–202. 10.1023/A:1015036617385

[21] Jensen HH. Consumer issues and demand. (2006). Available online at: https://www.choicesmagazine.org/2006-3/animal/2006-3-09.htm (accessed September 13, 2022).

[22] Poortinga W Pidgeon NF. Trust in risk regulation: cause or consequence of the acceptability of GM food? Risk Analysis. (2005) 25:199–209. 10.1111/j.0272-4332.2005.00579.x 15787769

[23] Etienne J Chirico S Gunabalasingham T Dautzenberg S Gysen S EU. Insights – Perceptions on the human health impact of antimicrobial resistance (AMR) and antibiotics use in animals across the EU. EFS3. (2017) 14:1183E. 10.2903/sp.efsa.2017.EN-1183

[24] Cornejo J Cabezón C Martín BS Lapierre L. Assessment of consumer perceptions on the use of antimicrobials in production animals in Chile. J Food Prot. (2018) 81:1331–8. 10.4315/0362-028X.JFP-17-463 30019962

[25] Clark B Panzone LA Stewart GB Kyriazakis I Niemi JK Latvala T. Consumer attitudes towards production diseases in intensive production systems. PLoS ONE. (2019) 14:e0210432. 10.1371/journal.pone.0210432 30629667 PMC6328233

[26] Goddard E Hartmann M Klink-Lehmann J. Public acceptance of antibiotic use in livestock production Canada and Germany. Proceed Food Sys Dyn. (2017) 3:424–37. 10.18461/PFSD.2017.1743

[27] Meeusen MJG Van den Berg I Voordouw J Van Haaster-de Winter M. Antibiotic use in Livestock Farming Through the Eyes of Consumers. Amsterdam (2014). in *3rd International Conference on Responsible Use of Antibiotics in Animals*, p. 101.

[28] Brewer MS Rojas M. Consumer attitudes toward issues in food safety. J Food Safety. (2008) 28:1–22. 10.1111/j.1745-4565.2007.00091.x

[29] Regan Á Sweeney S McKernan C Benson T Hyland J Dean M. The impact of the Covid-19 pandemic on food consumers' awareness of antimicrobial resistance, one health, and animal welfare information on food labels. Front Vet Sci. (2021) 8:678509. 10.3389/fvets.2021.678509 34268348 PMC8276886

[30] National Health Commission of the PRC. National Action Plan to Contain Antimicrobial Resistance (2016–2020). Available online at: http://en.nhc.gov.cn/2016-08/26/c_70276.htm (accessed August 23, 2022).

[31] FAO/WHO. Guidelines on Integrated Monitoring and Surveillance of Foodborne Antimicrobial Resistance. Available online at: https://www.fao.org/fao-who-codexalimentarius/meetings/detail/en/?meeting=TFAMR&session=8 (accessed August 24, 2022).

[32] FAO/WHO. Codex Guidelines on integrated monitoring and surveillance of food borne antimicrobial resistance and Code of Practice to minimize and contain food borne antimicrobial resistance. Available online at: https://www.amrleaders.org/resources/codex-guidelines-on-integrated-monitoring-and-surveillance-of-food-borne-antimicrobial-resistance-and-code-of-practice-to-minimize-and-contain-food-borne-antimicrobial-resistance (accessed August 24, 2022).

[33] Lusk JL Norwood FB Pruitt JR. Consumer demand for a ban on antibiotic drug use in pork production. Am J Agric Econ. (2006) 88:1015–33. 10.1111/j.1467-8276.2006.00913.x

[34] Powell D Chapman B. Risk communication during foodborne disease outbreaks: the four Rs. Foodborne Diseases: Case Studies of Outbreaks in the Agri-Food Industries. Boca Raton: CRC Press (2016). p. 383–404.

[35] Ritter GD Acuff GR Bergeron G Bourassa MW Chapman BJ Dickson JS. Antimicrobial-resistant bacterial infections from foods of animal origin: understanding and effectively communicating to consumers. Ann NY Acad Sci. (2019) 1441:40–9. 10.1111/nyas.14091 30924543 PMC6850152

