# Supplementary material for: Consumers' knowledge, attitude, and behavior towards antimicrobial resistance and antimicrobial use in food production in China
Source: Front Public Health. 2022 Sep 23;10:1015950. doi: 10.3389/fpubh.2022.1015950 (PMC9540231; doi:10.3389/fpubh.2022.1015950)
Supplement: Supplementary file 1 [file Data_Sheet_1.PDF]

Supplementary Material

Appendix: Survey Questionnaire Design

SECTIONS

- Screening questions.
- A: Knowledge and awareness of AMR and AMU in food production.
- B: Knowledge and awareness of current food regulations on AMU in China.
- C: Perceptions and attitudes towards AMU in food production and current food regulations on AMU in China.
- D: Behavioural changes and intentions.
- E: Communication and confidence in information sources.
- F: Demographic information.

INTRODUCTION

The following survey aims to investigate Chinese consumers' knowledge and attitudes on AMU in food animals and understand the behaviours and intentions that consumers make when purchasing animal-derived food products.

The questionnaire consists of 28 questions and will take you around 5-10 minutes to complete. All information collected during the survey will be kept strictly confidential. The data will be processed anonymously and you will not be personally identifiable in any research outputs or reports. There are no right and wrong answers. Please answer all the questions as openly and honestly as possible. You may leave the study at any time by closing your browser window if you would like to end the survey.

If you have any questions about this research, or would like to share anything with me, please contact me via email: dingqianyun11@zju.edu.cn

SCREENING QUESTIONS

Q1. I have read and understood the above instructions, and I give consent for my data to be included in this research.

Yes	1

Q2. Are you responsible for the food and grocery shopping (including online retailers) in your household?

Yes – I do most of the food and grocery shopping	1
Yes – I am jointly responsible / share responsibility with others	2
No – someone else does it	3

Q3. Approximately how often do you purchase animal-derived food products from the supermarket or other online retailers?

This includes every type of animal food products that may be purchased on their own or as part of a meal, such as pork, beef, lamb, poultry, milk, eggs, fish, etc.

Never	1
1-2 times daily	2

1-3 times a week	3
1-3 times a month	4
Every 2-3 months	5
1-3 times a year	6

SECTION A: KNOWLEDGE AND AWARENESS OF AMR AND AMU IN FOOD PRODUCTION

Q5. Have you ever taken antimicrobials before in your lifetime?

Yes	1
No	2

Q6. Have you had a situation where antimicrobials didn't work?

This means you were told by a medical professional that the antimicrobial didn't work.

Yes	1
No	2

Q7. Have you heard of antibiotic resistance or antimicrobial resistance (AMR)?

Yes	1
No	2

Q8. Do you know what AMR is?

Yes	1
No	2
Not sure	3

Q9. Please read the following statements and indicate if you think they are true or false.

No.		True	False	Don't Know/Not Sure
1	Antimicrobials kill viruses	1	2	3
2	Antimicrobials kill bacteria	1	2	3
3	Antimicrobials are used to cure infections in farm animals	1	2	3
4	Antimicrobials can stimulate the growth of farm animals	1	2	3
5	Antimicrobials are used more often to treat people rather than animals	1	2	3
6	Antimicrobials used on farm animals are different from those used on humans	1	2	3
7	Unnecessary use of antimicrobials in animals makes the antimicrobials become ineffective to treat animals	1	2	3
8	If food producing animals are treated with antimicrobials, then antimicrobials will be present in the meat	1	2	3
9	If I have AMR I will not be able to treat some illness	1	2	3

Correct Answer

Q10. Do you think that resistance to antimicrobials in animals may be transferred to humans?

No.		Yes	No	Don't Know/Not Sure
1	When humans handle raw meat	1	2	3
2	When humans eat lightly cooked meat (rare)	1	2	3
3	When humans eat well cooked meat	1	2	3
4	When humans come in contact with live farm animals	1	2	3
5	When they drink water that has been tainted by animal excrements	1	2	3
6	When they eat vegetables, cereals or fruits from soil that has been fertilized with animal excrements	1	2	3

Correct Answer

Q11. Do you think that AMR may be promoted under the following situations?

No.		Yes	No	Don't Know/Not Sure
1	Giving antimicrobials to healthy animals in order to prevent illness contributes to AMR	1	2	3
2	Giving antimicrobials to healthy animals in order to stimulate growth contributes to AMR	1	2	3
3	Treating unhealthy or weak animals with veterinary prescribed antimicrobials contributes to AMR	1	2	3
4	Treating unhealthy or weak animals without a veterinary prescription contributes to AMR	1	2	3

Correct Answer

SECTION B: KNOWLEDGE AND AWARENESS OF CURRENT FOOD REGULATIONS ON AMU IN CHINA

Q12. Please read the following statements and indicate if you think they are true or false.

No.		True	False	Don't Know/Not Sure
1	Resistance to antimicrobials is widespread in Chinese farming	1	2	3
2	All food products sold in China are regulated under Chinese law to ensure that they are safe for human consumption	1	2	3
3	Growth promoters are often used to stimulate rapid weight gain in food producing animals sold in China	1	2	3
4	Up to date, there are no regulations or restrictive provisions on antimicrobials used in food producing animals in China	1	2	3

Correct Answer

SECTION C: PERCEPTIONS AND ATTITUDES TOWARDS AMU IN FOOD PRODUCTION AND CURRENT FOOD REGULATIONS ON AMU IN CHINA

Q13. To what extent do you agree or disagree with each of the following statements?

Please use a scale of 1 to 5, where 1 means strongly disagree and 5 means strongly agree.

No.		Strongly disagree				Strongly agree
1	I am concerned that animal-derived food products I buy may contain antimicrobial residues	1	2	3	4	5
2	I am concerned that it may have an impact on my health to consume food products containing antimicrobial residues	1	2	3	4	5
3	I think it may have an impact on human health to come in contact with live farm animals which already have AMR	1	2	3	4	5
4	I think not enough actions have been undertaken to control or prevent overuse of antimicrobials in farm animals in China	1	2	3	4	5

SECTION D: BEHAVIOURAL CHANGES AND INTENTIONS

Q14. Has some of your following behaviours changed due to the concern of AMR and AMU in farm animals? (Multiple choices)

Change eating or cooking habits	1
Look for information about AMU on food packaging when purchasing food products	2
Talk to family or friends about AMR in farming	3
Talk to authorities or government about AMR	4
Change behaviour when contacting farm animals	5
No actions change	6
Others (Please supplement)	7

Q15. Are you willing to pay a higher price on antimicrobials-free food products certified by authorities?

Yes	1
No	2

(If answered 'yes', skip to Q17)

Q16. Why are you not willing to purchase antimicrobials-free food products? (Multiple choices)

Higher price	1
Certified authorities cannot be trusted	2
Antimicrobials-free food products may still contain antimicrobials residues	3
AMR may still be transferred to consumers	4
I think AMR in food producing animals may not impact human health	5
Others (Please supplement)	6

SECTION E: COMMUNICATION AND TRUST

Q17. Where did you notice this information about resistance to antimicrobials in farm animals? (Multiple choices)

Family and friends	1
Health professionals/Doctors	2

Veterinarians	3
Farmers	4
Scientists	5
Food companies/Supermarkets	6
National food safety agencies/Governments	7
Education from universities and academic institutions	8
Social training	9
Media (Newspaper, TV, radio)	10
Social media	11
Internet	12
Others (Please supplement)	13

Q18. How confident are you that the information about resistance to antimicrobials in farm animals provided by the following sources is accurate information?

No.		Strongly unconfident				Strongly confident
1	Family and friends	1	2	3	4	5
2	Health professionals/Doctors	1	2	3	4	5
3	Veterinarians	1	2	3	4	5
4	Farmers	1	2	3	4	5
5	Scientists	1	2	3	4	5
6	Food companies/Supermarkets	1	2	3	4	5
7	National food safety agencies/Governments	1	2	3	4	5
8	Education from universities and academic institutions	1	2	3	4	5
9	Social training	1	2	3	4	5
10	Media (Newspaper, TV, radio)	1	2	3	4	5
11	Social media	1	2	3	4	5
12	Internet	1	2	3	4	5

SECTION F: DEMOGRAPHICS INFORMATION

Q19. What is your age?

Under 18	1
18-30	2
31-40	3
41-50	4
51-60	5
61-80	6
80+	7

(Close under 18)

Q20. What is your gender?

Male	1
Female	2
Other	3

(Close for "other")

Q21. What is the highest level of education that you have completed?

Secondary education completed (Junior high school) and below	1
Secondary education completed (Senior high school)	2
College education completed	3
Bachelor education completed (first degree e.g. BA, BSc)	4
Postgraduate education completed and higher degree (e.g. Masters, doctorate, post-doctorate)	5

Q22. What is your work status?

Employed full-time (≥30 hours per week)	1
Employed part-time (≤29 hours per week)	2
Full-time homemaker	3
Student	4
Unemployed	5
Retired	6

Q23. Have you worked in any of the following occupations or been studying the major currently or in the past? (Multiple choices)

Agriculture, forestry, animal husbandry, fishing and water conservation	1
Industrial engineering	2
Geological survey and exploration	3
Construction	4
Transportation, posts and telecommunications	5
Commerce, public catering, supply and storage	6
Real estate management, public utilities, residential services and consulting services	7
Health, sports and social welfare	8
Education, culture, arts and broadcasting	9
Scientific research and integrated technical services	10
Finance and insurance	11
Government agencies and social organizations	12
None	13
Others (Please supplement)	14

Q24. What is the total income of your household from all sources before any tax and national insurance contributions? *If you share your household with individuals unrelated to you (not a family member or your partner), please count only your personal income. Include all income from employment and benefits.*

Under ¥ 50,000 per annum	1
¥ 50,000 - ¥ 100,000 per annum	2
¥ 100,000 - ¥ 200,000 per annum	3
¥ 200,000 - ¥ 500,000 per annum	4
¥ 500,000 - ¥ 1,000,000 per annum	5
¥ 1,000,000 + per annum	6
Not stable/Not sure	7
Prefer not to say	8

Q25. Including you, how many adults aged 18 or over live in your household?

1-2	1
3-5	2
5 +	3

Q26. How many children aged under 18 live in your household?

0	1
1-2	2
3 +	3

Q27. Where do you live? (Place of residence)

Beijing	1
Tianjin	2
Shanghai	3
Chongqing	4
Hebei	5
Shanxi	6
Liaoning	7
Jilin	8
Heilongjiang	9
Jiangsu	10
Zhejiang	11
Anhui	12
Fujian	13
Jiangxi	14
Shandong	15
Henan	16
Hubei	17
Hunan	18
Guangdong	19
Hainan	20
Sichuan	21

Guizhou	22
Yunnan	23
Shanxi	24
Gansu	25
Qinghai	26
Inner Mongoria	27
Guangxi	28
Tibet	29
Ningxia	30
Xinjiang	31
Hong Kong/Macao/Taiwan/Out of China	32

Q28. Is there anything else you would like to share regarding the study? Or do you have any suggestions for this survey?

Table S1. Demographic details and characteristics of the study sample. (N = 1065)

**Demographic Group**	Frequency (N)	Percentage (%)
**Gender**		
Male	399	37.5%
Female	666	62.5%
**Age**		
18-30	646	60.7%
31-40	180	16.9%
41-50	109	10.2%
51-60	80	7.5%
61-80	41	3.8%
80+	9	0.8%
**Highest education level**		
Secondary education completed (Junior high school) and below	34	3.2%
Secondary education completed (Senior high school)	126	11.8%
College education completed	248	23.3%
Bachelor education completed	533	50.0%
Postgraduate education completed and higher degree	124	11.6%
**Work status**		
Employed full-time	617	57.9%
Employed part-time	38	3.6%
Full-time homemaker	32	3.0%
Student	266	25.0%
Unemployed	36	3.4%
Retired	76	7.1%
**Occupation**		
Agriculture, forestry, animal husbandry, fishing, and water conservation	105	9.9%
Industrial engineering	121	11.4%
Geological survey and exploration	20	1.9%
Construction	66	6.2%
Transportation, posts, and telecommunications	62	5.8%
Commerce, public catering, supply, and storage	113	10.6%
Real estate management, public utilities, residential services, and consulting services	90	8.5%
Health, sports, and social welfare	173	16.2%
Education, culture, arts, and broadcasting	214	20.1%
Scientific research and integrated technical services	70	6.6%
Finance and insurance	88	8.3%
Government agencies and social organizations	62	5.8%
None	96	9.0%
**Household income**		
Under ¥ 50,000 per annum	137	12.9%

¥ 50,000 - ¥ 100,000 per annum	282	26.5%
¥ 100,000 - ¥ 200,000 per annum	269	25.3%
¥ 200,000 - ¥ 500,000 per annum	167	15.7%
¥ 500,000 - ¥ 1,000,000 per annum	61	5.7%
¥ 1,000,000 + per annum	10	0.9%
Not stable/Not sure	45	4.2%
Prefer not to say	94	8.8%
**Household adults**		
1-2	356	33.4%
3-5	622	58.4%
5 +	87	8.2%
**Household children**		
0	504	47.3%
1-2	527	49.5%
3 +	34	3.2%
**Place of residence**		
Beijing	45	4.2%
Tianjin	9	0.8%
Shanghai	46	4.3%
Chongqing	51	4.8%
Hebei	45	4.2%
Shanxi	27	2.5%
Liaoning	20	1.9%
Jilin	14	1.3%
Heilongjiang	18	1.7%
Jiangsu	52	4.9%
Zhejiang	335	31.5%
Anhui	31	2.9%
Fujian	18	1.7%
Jiangxi	22	2.1%
Shandong	35	3.3%
Henan	54	5.1%
Hubei	30	2.8%
Hunan	24	2.3%
Guangdong	92	8.6%
Hainan	2	0.2%
Sichuan	28	2.6%
Guizhou	13	1.2%
Yunnan	3	0.3%
Shanxi	21	2.0%
Gansu	4	0.4%
Qinghai	0	0.0%

Inner Mongoria	4	0.4%
Guangxi	12	1.1%
Tibet	0	0.0%
Ningxia	1	0.1%
Xinjiang	7	0.7%
Hong Kong/Macao/Taiwan/Out of China	2	0.2%

Table S2. Sum scores from Q9 to Q11, expressed as Mean±SD (95%CI), were performed by ANOVA test and Bonferroni post-hoc test compared with "highest education level", "work status", and "household income". (N = 1065)

Demographic Group	Sum scores of knowledge	*F* value	*P* value
**Highest education level**		**16.3**	**<0.001**
Secondary education completed (Junior high school) and below	6.8±4.8 (5.0-8.3)		<0.001[ab]
Secondary education completed (Senior high school)	7.7±3.7 (7.0-8.3)		<0.001[ab]
College education completed	7.9±3.7 (7.5-8.4)		<0.001[ab]
Bachelor education completed [a]	9.3±3.5 (9.0-9.6)		0.044[b]
Postgraduate education completed and higher degree [b]	10.3±3.3 (9.7-10.9)		------
**Work status**		**3.9**	**0.002**
Employed full-time [c]	9.1±3.6 (8.9-9.4)		------
Employed part-time	8.4±4.3 (7.0-9.8)		------
Full-time homemaker	8.4±4.3 (7.1-9.6)		------
Student	8.6±3.7 (8.2-9.1)		------
Unemployed	6.9±3.9 (5.6-8.3)		0.008 [c]
Retired	8.0±4.0 (7.1-8.9)		------
**Household income**		**5.6**	**<0.001**
Under ¥ 50,000 per annum	8.0±4.0 (7.3-8.7)		<0.001[d]
¥ 50,000 - ¥ 100,000 per annum	8.6±3.6 (8.2-9.0)		0.007[d]
¥ 100,000 - ¥ 200,000 per annum	9.0±3.5 (8.6-9.4)		------
¥ 200,000 - ¥ 500,000 per annum [d]	9.9±3.2 (9.4-10.4)		------
¥ 500,000 - ¥ 1,000,000 per annum [e]	9.8±4.0 (8.7-10.8)		------
¥ 1,000,000 + per annum	8.7±3.4 (6.3-11.1)		------
Not stable/Not sure	8.1±3.9 (6.9-9.2)		------
Prefer not to say	6.9-8.5		<0.001[d], 0.014[e]

The groups whose *P* value >0.05 was not presented in the table.

[a] Statistical significance compared with "Bachelor education completed" group in education.

[b] Statistical significance compared with "Postgraduate education completed and higher degree" group in education.

[c] Statistical significance compared with "Employed full-time" group in work status.

[d] Statistical significance compared with "¥ 200,000 - ¥ 500,000 per annum" group in household income.

[e] Statistical significance compared with "¥ 500,000 - ¥ 1,000,000 per annum" group in household income.

Table S3. Mean scores of the first three and the fourth statements in Q13, expressed as Mean±SD (95%CI), were performed by ANOVA test and Bonferroni post-hoc test compared with "age", "highest education level", "work status", and "Household income". (N = 1065)

Demographic Group	Scale of antimicrobial-use attitude			Scale of China-situation attitude		
	Mean±SD (95%CI)	ANOVA test		Mean±SD (95%CI)	ANOVA test	
		F value	P value		F value	P value
**Age**		**9.2**	**<0.001**		**5.3**	**<0.001**
18-30 [a]	3.4±1.0 (3.3-3.5)		------	2.8±1.1 (2.7-2.8)		------
31-40 [b]	3.8±1.0 (3.7-4.0)		<0.001[a]	2.8±1.3 (2.6-3.0)		------
41-50	3.9±1.1 (3.7-4.1)		<0.001[a]	3.2±1.4 (3.0-3.5)		<0.001[a]
51-60	3.8±1.0 (3.6-4.0)		0.038[a]	3.3±1.3 (3.0-3.6)		<0.001[a], 0.035[b]
61-80	3.8±1.1 (3.4-4.1)		------	3.2±1.3 (2.8-3.6)		------
80+	3.2±1.3 (2.2-4.2)		------	3.3±1.6 (2.1-4.5)		------
**Highest education level**		**2.6**	**0.038**		**5.3**	**<0.001**
Secondary education completed (Junior high school) and below			------	2.8±1.4 (2.3-3.3)		------
Secondary education completed (Senior high school)			------	2.8±1.3 (2.5-3.0)		0.002[c]
College education completed			------	2.8±1.1 (2.6-2.9)		<0.001[c]
Bachelor education completed			------	2.9±1.2 (2.8-3.0)		0.001[c]
Postgraduate education completed and higher degree [c]			------	3.3±1.1 (3.1-3.5)		------
**Work status**		**3.0**	**0.010**		**2.6**	**0.030**
Employed full-time	3.7±1.0 (3.6-3.7)		0.005[d]	2.9±1.2 (2.8-3.0)		------
Employed part-time	3.5±1.0 (3.1-3.8)		------	2.8±1.2 (2.4-3.2)		------
Full-time homemaker	3.8±1.1 (3.4-4.2)		------	2.8±1.4 (2.3-3.3)		------
Student [d]	3.4±1.0 (3.3-3.5)		------	2.7±1.0 (2.6-2.8)		------
Unemployed	3.5±0.9 (3.2-3.8)		------	3.0±1.1 (2.6-3.3)		------
Retired	3.7±1.1 (3.4-3.9)		------	3.2±1.4 (2.9-3.5)		0.028[d]
**Household income**		**3.0**	**0.005**		**3.8**	**<0.001**
Under ¥ 50,000 per annum	3.4±1.0 (3.3-3.6)		0.026[e]	2.8±1.1 (2.6-3.0)		------
¥ 50,000 - ¥ 100,000 per annum	3.5±1.0 (3.4-3.6)		------	2.7±1.1 (2.6-2.8)		0.003[e], 0.015[f]

¥ 100,000 - ¥ 200,000 per annum	3.5±1.0 (3.4-3.6)	0.033[e]	2.9±1.1 (2.8-3.1)	------
¥ 200,000 - ¥ 500,000 per annum [e]	3.8±1.0 (3.6-4.0)	------	3.2±1.3 (3.0-3.4)	------
¥ 500,000 - ¥ 1,000,000 per annum [f]	3.8±1.1 (3.5-4.1)	------	3.3±1.4 (2.9-3.6)	------
¥ 1,000,000 + per annum	3.5±0.9 (2.8-4.1)	------	3.4±1.3 (2.5-4.3)	------
Not stable/Not sure	3.6±1.1 (3.3-4.0)	------	2.5±1.2 (2.2-2.9)	0.027[e], 0.019[f]
Prefer not to say	3.7±1.0 (3.5-3.9)	------	2.8±1.3 (2.5-3.1)	------

The groups whose *P* value >0.05 were not presented in the table.

[a] Statistical significance compared with “18-30” group of age.

[b] Statistical significance compared with “31-40” group of age.

[c] Statistical significance compared with “Postgraduate education completed and higher degree” group of education.

[d] Statistical significance compared with “Student” group of work status.

[e] Statistical significance compared with “¥ 200,000 - ¥ 500,000 per annum” group of household income.

[f] Statistical significance compared with “¥ 500,000 - ¥ 1,000,000 per annum” group of household income.

Table S4. Percentage of willingness to pay were performed by Chi-square test compared with "age", "work status", and "household income". (N = 1065)

Demographic Group	Total (N)	Willingness to pay Answered "Yes" N(%)	χ^2 Test	
			χ^2 value	P value
**Age**	**1065**	**850(79.8%)**	**32.9**	**<0.001**
18-30 [a]	646	482(74.6%)		------
31-40	180	155(86.1%)		0.001[a]
41-50	109	97(89.0%)		0.001[a]
51-60	80	74(92.5%)		<0.001[a]
61-80	41	36(87.8%)		------
80+	9	6(66.7%)		------
**Work status**			**21.8**	**<0.001**
Employed full-time	617	513(83.1%)		<0.001[b]
Employed part-time	38	31(81.6%)		------
Full-time homemaker	32	26(81.3%)		------
Student [b]	266	189(71.1%)		------
Unemployed	36	25(69.4%)		------
Retired	76	66(86.8%)		------
**Household income**			**40.9**	**<0.001**
Under ¥ 50,000 per annum	137	93(67.9%)		<0.001[cd]
¥ 50,000 - ¥ 100,000 per annum	282	224(79.4%)		0.001[d]
¥ 100,000 - ¥ 200,000 per annum [c]	269	226(84.0%)		------
¥ 200,000 - ¥ 500,000 per annum [d]	167	152(91.0%)		------
¥ 500,000 - ¥ 1,000,000 per annum	61	52(85.2%)		------
¥ 1,000,000 + per annum	10	7(70.0%)		------
Not stable/Not sure	45	33(73.3%)		0.002[d]
Prefer not to say	94	63(67.0%)		<0.001[cd]

The groups whose *P* value >0.05 were not presented in the table.

[a] Statistical significance compared with "18-30" group of age.

[b] Statistical significance compared with "Student" group of work status.

[c] Statistical significance compared with "¥ 100,000 - ¥ 200,000 per annum" group of household income.

[d] Statistical significance compared with "¥ 200,000 - ¥ 500,000 per annum" group of household income.

Table S5. Percentage and Mean±SD of scales of confidence in information sources (N = 1065).

Scale	Information sources	Strongly unconfident N (%)	Unconfident N (%)	Neutral/ Don't know/ Not sure N (%)	Confident N (%)	Strongly confident N (%)	Mean±SD (95% CI)
**Professionals and authorities**	Scientists	23(2.2%)	49(4.6%)	187(17.6%)	335(31.5%)	471(44.2%)	4.1±1.0(4.1-4.2)
	Health professionals/Doctors	16(1.5%)	43(4.0%)	202(19.0%)	384(36.1%)	420(39.4%)	4.1±0.9(4.0-4.1)
	National food safety agencies/Governments	45(4.2%)	66(6.2%)	259(24.3%)	321(30.1%)	374(35.1%)	3.9±1.1(3.8-3.9)
	Veterinarians	31(2.9%)	81(7.6%)	298(28.0%)	352(33.1%)	303(28.5%)	3.8±1.0(3.7-3.8)
	Education from universities and academic institutions	26(2.4%)	77(7.2%)	324(30.4%)	352(33.1%)	286(26.9%)	3.8±1.0(3.7-3.8)
**Media, Internet, word of mouth, others**	Family and friends	34(3.2%)	116(10.9%)	431(40.5%)	282(26.5%)	202(19.0%)	3.5±1.0(3.4-3.5)
	Media (Newspaper, TV, radio)	53(5.0%)	133(12.5%)	455(42.7%)	285(26.8%)	139(13.1%)	3.3±1.0(3.2-3.4)
	Social training	72(6.8%)	167(15.7%)	452(42.4%)	260(24.4%)	114(10.7%)	3.2±1.0(3.1-3.2)
	Social media	70(6.6%)	169(15.9%)	488(45.8%)	219(20.6%)	119(11.2%)	3.1±1.0(3.1-3.2)
	Food companies/Supermarkets	77(7.2%)	165(15.5%)	493(46.3%)	228(21.4%)	102(9.6%)	3.1±1.0(3.1-3.2)
	Internet	93(8.7%)	171(16.1%)	473(44.4%)	230(21.6%)	98(9.2%)	3.1±1.0(3.0-3.1)
	Farmers	102(9.6%)	204(19.2%)	477(44.8%)	175(16.4%)	107(10.0%)	3.0±1.1(3.9-3.0)

(1=strongly unconfident, 2=unconfident, 3=neutral/don't know/not sure, 4=confident, 5=strongly confident)
